# Crosstalk Effects in a Dual ToF-Based Tactile–Proximity Sensing Platform Integrated in a Flat PMMA Light Guide

**DOI:** 10.3390/s25237319

**Published:** 2025-12-02

**Authors:** Andrejs Ogurcovs, Ilze Aulika, Sergio Cartiel, Jorge Garcia-Pueyo, Adolfo Muñoz

**Affiliations:** 1 Institute of Solid State Physics, University of Latvia, Kengaraga Street 8, LV-1063 Riga, Latvia; ilze.aulika@cfi.lu.lv; 2 Instituto de Investigación en Ingeniería de Aragón (I3A) Zaragoza, University of Zaragoza, C. Maria de Luna, 1, 50019 Zaragoza, Spain; scartiel@unizar.es (S.C.); jorge.garciap@unizar.es (J.G.-P.); adolfo@unizar.es (A.M.)

**Keywords:** ToF sensor, TMF8828, acrylic glass, distance measurements, OptoSkin, optical transduction, tactile sensing, sensor interference, proximity

## Abstract

We investigate crosstalk effects in a dual-modality tactile–proximity sensing system based on Time-of-Flight (ToF) technology integrated within a flat poly(methyl methacrylate) (PMMA) light guide. Building on the OptoSkin framework, we employ two commercially available TMF8828 multi-zone ToF sensors, one configured for tactile detection via frustrated total internal reflection (FTIR) and the other for external proximity measurements through the same transparent substrate. Controlled experiments were conducted using a 2 cm^2^ silicone pad for tactile interaction and an A4-sized diffuse white target for proximity detection. Additional measurements with a movable PMMA sheet were performed to quantify signal attenuation, peak broadening, and confidence degradation under transparent-substrate conditions. The results demonstrate that the TMF8828 can simultaneously resolve both contact-induced scattering and distant reflections, but that localized interference zones occur when sensor fields of view overlap within the substrate. Histogram analysis reveals the underlying multi-path contributions, providing diagnostic insight not available from black-box ToF devices. These findings highlight both the opportunities and limitations of integrating multiple ToF sensors into transparent waveguides and inform design strategies for scalable robotic skins, wearable interfaces, and multi-modal human–machine interaction systems.

## 1. Introduction

Tactile and proximity sensing are foundational capabilities for intelligent robotic systems, wearable devices, and collaborative automation. These modalities enable machines to perceive and respond to physical contact, detect nearby objects, and operate safely around humans. As human–machine interaction becomes more prevalent in areas such as rehabilitation, smart prosthetics, soft robotics, and collaborative manufacturing, integrating multimodal sensing into compact, robust, and transparent interfaces has become a critical design goal [1,2]. Conventional range-sensing solutions—such as external cameras, structured-light systems, or scanning LiDAR units—provide high-resolution environmental awareness but are often unsuitable for close-range or embedded applications. Cameras and vision-based tactile sensors can capture detailed contact and proximity information but generally depend on line-of-sight, suffer from occlusions and lighting variability, and often rely on relatively bulky optics or embedded imagers [3,4]. Scanning LiDARs and other macroscopic ranging systems, while accurate, are power-intensive and primarily designed as stand-alone modules for environment mapping, which makes them difficult to embed into soft or conformal surfaces [5]. Capacitive and resistive tactile sensors provide lightweight contact detection but are often limited to 2D surfaces and require dense electrode arrays or multilayered construction, complicating large-scale integration [6].

Compact time-of-flight (ToF) sensors offer an attractive alternative. These modules detect surface deformation and object proximity with millimeter precision and are available in miniaturized packages suitable for embedded platforms [7]. ToF sensors emit modulated infrared light and measure the return time of reflected photons to infer distance. When positioned behind transparent substrates, they can support both proximity and tactile sensing—if optical coupling is well managed. In prior work, Bacher et al. introduced OptoSkin [1], a tactile sensing platform using direct ToF sensors embedded in transparent waveguides. It leveraged frustrated total internal reflection (FTIR) in poly(methyl methacrylate) (PMMA), where light propagation is disrupted by surface contact, enabling touch detection via scattered photon return. The TMF8828 sensor captured these signals with low latency and multi-zone resolution, enabling contact sensing without external cameras or electrodes. However, this configuration was limited to contact detection and lacked anticipatory (proximity) capabilities. In this context, we distinguish between macroscopic scanning LiDAR systems for long-range three-dimensional mapping and compact direct ToF (dToF) modules such as the TMF8828. Both rely on the same physical time-of-flight principle, but dToF modules are optimized for low-power, embedded operation and are the focus of the present work.

Building on this foundation, we investigate a dual-sensor system that combines FTIR-based tactile sensing and external proximity detection within the same PMMA layer using two TMF8828 modules. By co-locating multiple ToF sensors in a shared substrate, we aim to create a compact, multifunctional interface for robotic skins, wearable surfaces, and interactive displays. However, embedding multiple active emitters behind transparent media introduces challenges. Optical interference, reflection artifacts, and cross-sensor crosstalk can degrade signal quality. Transparent materials like PMMA (refractive index ∼1.49) cause internal reflections and partial scattering, especially in the presence of surface roughness or air gaps [8]. Overlapping fields of view, particularly when sensors operate at the same wavelength (940 nm), can lead to signal amplification or masking effects that compromise measurement integrity. Crosstalk and multipath interference in ToF systems have been extensively analyzed for free-space cameras and LiDARs, and are often mitigated through modulation coding, optical design, or model-based reconstruction [9,10]. These studies, however, primarily consider imaging configurations or long-range sensing and do not address the case in which multiple dToF modules inject and collect light within the same transparent light guide that simultaneously serves as a tactile transducer.

**Figure 1:**
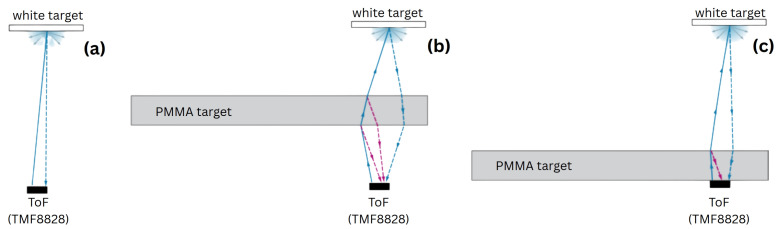
Schematic illustration of the experimental configurations for ToF measurements with a PMMA obstacle: (**a**) Baseline case: Distance measured to a white diffuse target without any PMMA obstacle. (**b**) PMMA positioned within the optical path: Both the PMMA surface and the distant white target contribute detectable reflections, leading to dual-peak histogram responses. (**c**) PMMA placed directly in front of the sensor: PMMA reflection dominates, while the white target peak is weakened or reassigned depending on the relative signal strength.

To explore these effects, we conducted controlled experiments with two TMF8828 sensors embedded in a shared PMMA light guide. One sensor was configured for FTIR-based tactile detection using a small silicone pad as the contact object, while the second sensor simultaneously measured distance to an external white target (Figure 1). Additional measurements with a movable PMMA sheet were performed to quantify substrate-induced attenuation. These experiments enabled us to characterize interference zones, quantify signal perturbations, and assess the scalability of such dual-modality systems.

Although capacitive [6], resistive [6], and optical [11,12] proximity systems have been extensively studied, a large portion of the existing work on optical skins and FTIR-based touch interfaces relies on camera-based imaging or single sensing modalities, including visuotactile fingertips such as FingerVision and dual-sided FTIR touch panels [4,13]. Similarly, the ToF literature has characterized multipath and crosstalk phenomena mainly in free-space imaging configurations rather than in embedded light-guide geometries [5,9,10]. Few works address the co-integration of ToF-based tactile and proximity sensing within the same transparent interface. Our work addresses this gap by systematically evaluating cross-sensor interference and transparent-substrate effects in a unified experimental framework. This contribution is relevant to developers of intelligent robotic skins, transparent HMI surfaces, and embedded sensing systems requiring compact, camera-free, multimodal feedback.

## 2. Materials and Methods

### 2.1. Sensor Platform (TMF8828)

All experiments in this work were carried out with the TMF8828 time-of-flight (ToF) module (ams-OSRAM AG, Premstätten, Austria). The device integrates a 940 nm VCSEL emitter and a SPAD receiver array and operates in a multi-zone configuration, providing per-zone range information together with the underlying raw histogram data. Unlike “black-box” ToF sensors that provide only a single distance estimate, the TMF8828 exposes the photon-count histograms for each zone via I^2^C, which enables transparent analysis of attenuation, multipath, crosstalk, and algorithm behavior (for more information on TMF8828 please refer to official manufacturer’s documentation [14]). The sensor supports up to 8 × 8 zones per measurement, where the zones are defined by an internal SPAD mask and can be reconfigured for different angular coverages. Each zone outputs a 128-bin photon-count histogram along with distance and confidence values, and the device can register up to two returns per zone when multiple reflectors are present. Measurement parameters such as timing budget, histogram averaging, and zone masks are configurable, which allows a trade-off between signal-to-noise ratio, update rate, and angular resolution. For the experiments described in this work, the TMF8828 was operated in short-range, a high-accuracy mode to maximize sensitivity to small signal changes caused by transparent substrates and FTIR-coupled touch events. In the following, we use the term *sensing platform* to denote the combination of the TMF8828 module, the transparent PMMA light guide, and the associated data-processing pipeline, rather than a single integrated chip.

All measurements were recorded in 8 × 8 multi-zone mode with 128-bin histograms enabled. In post-processing, the raw histograms together with the device-reported distance and confidence were used to quantify peak amplitudes, perform baseline subtraction, detect multiple returns, and visualize per-zone responses in polar or Cartesian reconstructions. This level of data access proved crucial for distinguishing the responses of a transparent PMMA sheet and a distant diffuse target, and for diagnosing crosstalk effects in FTIR touch configurations. For detailed insight into the algorithmic pipeline and mathematical formulation used for touch point reconstruction from raw histogram data, readers are referred to our earlier work [15], which presents an in-depth analysis of FTIR-based signal formation, calibration routines, and multi-point localization strategies. In the present study, this touch-point reconstruction algorithm is used without modification: all tactile information is obtained from the FTIR-coupled TMF8828 module, while proximity information is taken from the standard range and confidence outputs of the second TMF8828 module. The proximity channel is processed independently from the tactile reconstruction, and no sensor-fusion step combining the two modalities is applied. Consequently, any influence of the proximity measurements on the tactile output (and vice versa) arises purely from optical crosstalk in the shared PMMA guide rather than from changes in the algorithm. The current study extends those findings by integrating proximity and tactile modalities within a unified sensing architecture and experimentally examining inter-sensor optical interference.

### 2.2. Experimental Setup with Transparent PMMA

To evaluate the effect of transparent substrates on the TMF8828 ToF response, a series of measurements were conducted using a 2 mm thick PMMA sheet (SIA Ultraplast EU, Ventspils, Latvia, CAS No.: 9011-14-7) of A4 dimensions as an intermediate optical element. The PMMA plate was positioned between the ToF sensor and a matte white A4-size printer-paper sheet used as a diffuse reference target placed at a fixed distance of 300 mm from the sensor (Figure 1). The PMMA was mounted on a translation stage and moved along the optical axis in 10 mm increments, starting from direct contact with the sensor aperture and extending up to 280 mm.

At each step, the TMF8828 operated in multi-zone mode and produced raw photon-count histograms together with zone-specific distance and confidence values. From the recorded data, the histogram maximum values, mean distance, and mean confidence were extracted. Particular attention was given to the first and second return peaks, since the device can register multiple reflectors simultaneously (i.e., the PMMA interface and the distant paper target).

The acquired results were compiled into summary graphs showing peak intensity, mean distance, and mean confidence as a function of PMMA position. In addition, individual histogram curves at representative positions were analyzed separately to highlight the evolution of signal attenuation, peak broadening, and the emergence or suppression of multiple returns. This approach enabled a systematic assessment of how the transparent PMMA substrate perturbs the ToF signal and provided deeper insight into the limitations and opportunities of FTIR-based touch sensing when combined with a multi-zone ToF platform.

### 2.3. Experimental Setup with Dual-Sensor Layout

**Figure 2:**
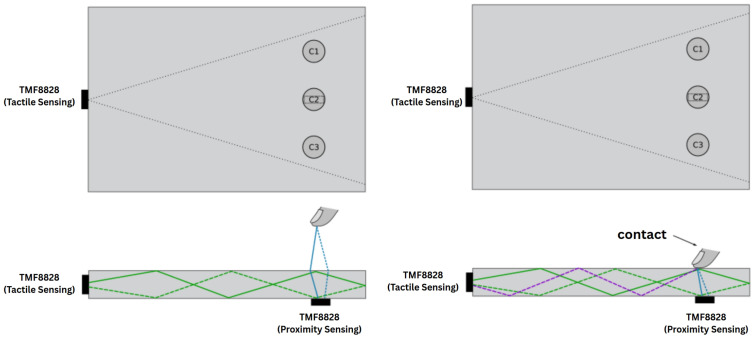
Top and side views of the experimental setup for dual ToF-based tactile and proximity sensing with two TMF8828 sensors coupled to a PMMA light guide. The tactile sensor is edge-mounted for FTIR-based touch detection, whereas the proximity sensor is located beneath the surface at position C2 and measures vertical distance through the same substrate. Contact points C1–C3 indicate the tested locations of the silicone pad relative to the proximity sensor.

To investigate crosstalk effects between two TMF8828 sensors in a combined tactile–proximity configuration, a transparent PMMA light guide was prepared with one sensor coupled to its edge for FTIR-based touch detection and a second sensor positioned at a neighboring edge for proximity measurements. Both sensors operated in multi-zone, short-range mode with histogram acquisition enabled. In the remainder of this section, we refer to the edge-mounted module as the *tactile sensor* and to the module located beneath the surface at position C2 as the *proximity sensor*. Three contact locations, denoted C1–C3 in Figure 2, were defined along the light guide to probe how the relative distance between the contact point and the proximity sensor influences the observed crosstalk.

For the tactile interaction, a small 2 cm^2^ silicone pad was pressed against the surface of the PMMA light guide directly above the first sensor’s field of view. This produced localized scattering and allowed the FTIR mechanism to be examined under controlled conditions. During each trial, the pad was placed successively at positions C1, C2, and C3, corresponding to regions farther from, directly above, and slightly beyond the proximity sensor, respectively.

For the proximity measurement, a diffuse white A4-size paper target was positioned above the light guide in alignment with the second TMF8828 sensor’s field of view. The distance to the target was fixed, ensuring stable external reflections while the tactile events were introduced (Figure 2).

During the experiment, both sensors acquired raw histograms, distance values, and confidence metrics simultaneously. The setup enabled the observation of how scattering introduced by the silicone–PMMA contact influenced the external distance measurement, and conversely, how the presence of the distant target affected the histogram structure of the FTIR-coupled channel. These conditions provided a controlled framework for evaluating optical crosstalk between tactile and proximity modalities using two identical TMF8828 sensors.

## 3. Results and Discussion

### 3.1. Setup with Transparent PMMA

**Figure 3:**
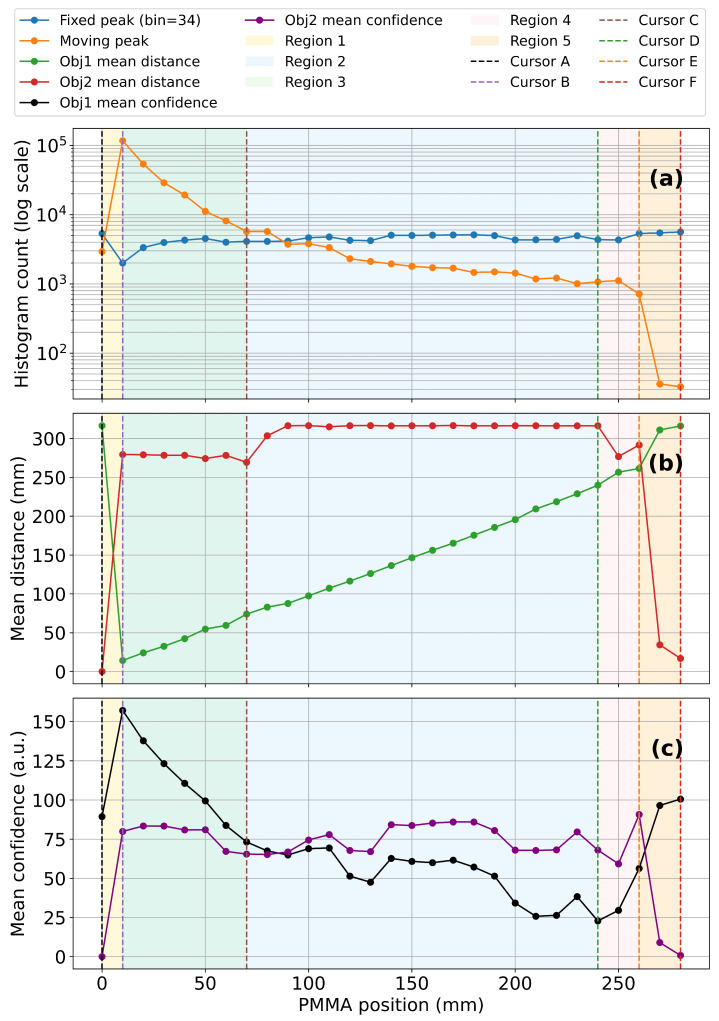
Influence of PMMA light guide position on ToF sensor performance: (**a**) Peak intensities as a function of PMMA position, showing the evolution of histogram counts (log scale) for the moving PMMA peak (Obj1) and the fixed far-target peak (Obj2) across five characteristic regions. (**b**) Mean distance readings versus PMMA position, highlighting deviations of the detected target from the 315 mm baseline and the reassignment of target/PMMA reflections between first and second object slots. (**c**) Mean confidence values versus PMMA position for both objects, illustrating the stability of the PMMA reflection and the reduced confidence of the target signal in regions dominated by parasitic reflections.

**Figure 4:**
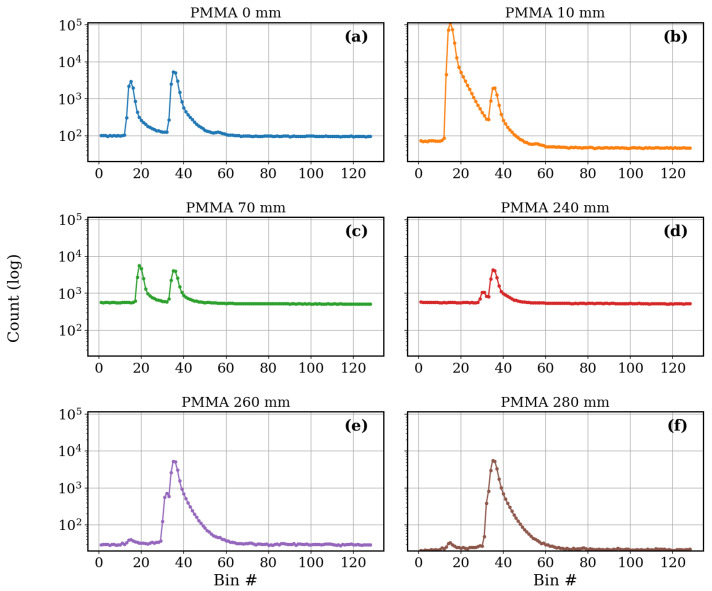
Representative ToF histogram responses at selected PMMA positions relative to the sensor aperture: (**a**) PMMA at 0 mm: Clear separation between PMMA reflection (Obj1) and target peak (Obj2). (**b**) PMMA at 10 mm: PMMA reflection dominates, causing reassignment of the target to the second object slot. (**c**) PMMA at 70 mm: Stable dual-peak response with well-separated PMMA and target signals. (**d**) PMMA at 240 mm: Target reassigned to the first object, while PMMA reflection shifts to weaker secondary peak. (**e**) PMMA at 260 mm: Unstable behavior with strong overlap between PMMA and target reflections. (**f**) PMMA at 280 mm: Only the target peak remains distinguishable, while PMMA reflection is suppressed.

To investigate the influence of transparent substrates on the measurement characteristics of the TMF8828, a 2 mm thick PMMA sheet was mounted on a linear translation stage and displaced from 0 to 280 mm along the optical axis. At each position, histogram frames were recorded to extract the main parameters of the detected peaks—namely peak intensity, mean distance, and confidence. Owing to its ability to report two peaks simultaneously, the TMF8828 could separately resolve the reflection from the PMMA sheet (moving peak) and the white reference target placed at 315 mm (fixed peak). This provides considerably more insight into the optical interaction between the propagating beam and the transparent obstacle compared to sensors that only output a single scalar distance value. Throughout this subsection, we refer to the PMMA-related return as Obj1 and to the distant diffuse target as Obj2; these labels are used consistently in the legends of Figure 3 and Figure 4.

The results are summarized in Figure 3. In the legend and subsequent discussion, Obj1 denotes the return from the PMMA sheet and Obj2 denotes the return from the distant paper target. The top panel (Figure 3a) shows the evolution of peak intensities on a logarithmic scale. Upon insertion of the PMMA sheet into the sensor field of view, a distinct moving peak appeared with high amplitude. As the sheet advanced deeper into the optical path, the intensity of this peak gradually decayed, spanning nearly three orders of magnitude across the full displacement range. In contrast, the fixed peak associated with the far reflector remained relatively stable, showing only moderate attenuation due to scattering and absorption within the PMMA.

The middle panel (Figure 3b) presents the evolution of mean distances. Without PMMA, the baseline distance to the white target was 315 mm. At 0 mm displacement, the measured value was 316 mm, fully consistent with the intrinsic accuracy of the sensor. However, once the PMMA was shifted 10 mm away from the aperture, the system exhibited an object swap: the PMMA reflection became the first object, while the white target was reassigned as the second. In this case, the mean distance reading for the target dropped to 279 mm, a deviation of approximately 13% from the baseline. Between 10–70 mm displacement, this behavior remained stable, with the white target consistently reported as the second object and distance readings clustering around 278 mm. From 70–240 mm, the target signal gradually regained dominance, and the measured distance steadily converged back to 315–316 mm, recovering the baseline accuracy. Beyond 240 mm, however, a transition region emerged: between 240–260 mm the histogram exhibited strong fluctuations, with alternating dominance of the target and PMMA peaks, leading to unstable distance readings. Finally, in the 260–280 mm range, the behavior resembled the initial 0–10 mm interval but in reverse: the white target was reassigned as the first object, restoring accurate readings of 315 mm, while the PMMA reflection collapsed.

The bottom panel (Figure 3c) shows the corresponding confidence values. The confidence of the moving PMMA peak was initially high but decreased steadily with insertion depth, reflecting the decay in its intensity. The target peak maintained high confidence over most of the displacement range, only collapsing sharply in the 240–260 mm transition zone. Importantly, the distance assigned to the PMMA itself remained highly accurate—within ±1 mm—up to 260 mm, demonstrating the robustness of the TMF8828 in resolving both a transparent layer and a fixed background target.

For clarity, the experiment can thus be divided into five operating regions: (1) 0–10 mm, where near and far peaks overlap strongly; (2) 10–70 mm, where the PMMA reflection dominates and the target is shifted to second object with 13% deviation; (3) 70–240 mm, where the target steadily recovers as the dominant peak; (4) 240–260 mm, an unstable transition with competing reflections; and (5) 260–280 mm, where the target regains priority and accurate baseline readings are restored.

To gain deeper insight into how transparent dielectric substrates influence the raw photon distribution, histogram data from the TMF8828 was analyzed at representative PMMA positions across the sensor’s field of view (Figure 4). These histograms complement the average curves shown in Figure 3 and allow the dynamics of peak evolution to be visualized directly.

At the initial position of 0 mm (Figure 4a), two well-defined peaks are observed. The first corresponds to the PMMA sheet, which introduces a strong scattering contribution at short distances, while the second represents the fixed reflective target located deeper in the measurement volume. The separation between the two peaks is stable, confirming the capability of the multi-zone ToF device to distinguish multiple optical paths. When the PMMA is shifted to 10 mm (Figure 4b), the intensity of its associated peak becomes dominant, while the fixed reference peak is still visible but already shows signs of attenuation. This indicates that even at very shallow insertion depths, the PMMA efficiently couples and redirects part of the guided infrared light, redistributing the photon counts away from the background reference. At 70 mm (Figure 4c), both peaks remain clearly separable, but the PMMA-induced peak has started to decay exponentially. This attenuation reflects the absorption and scattering losses accumulated along the increasing optical path length in the transparent substrate. The fixed peak is still detectable at this stage, albeit with lower amplitude, demonstrating the dual-object detection capacity of the sensor. By 240 mm (Figure 4d), the moving PMMA peak is still present but its amplitude has decreased by nearly two orders of magnitude compared to the 10 mm case. The histogram also begins to show overlap between the decaying PMMA peak and the fixed reference peak, highlighting the challenge of distinguishing multiple transparent objects at longer propagation distances. At 260 mm (Figure 4e), the interference becomes pronounced. Both peaks broaden and partially merge, producing distorted shapes and unstable centroid positions. This corresponds to the transition region identified in the averaged confidence metrics (Figure 3c), where object recognition reliability rapidly deteriorates. Finally, at 280 mm (Figure 4f), only a faint residual of the fixed reference peak remains. The PMMA-associated contribution has essentially disappeared from the histogram, confirming that the transparent substrate has exited the effective sensing volume. Taken together, these representative histograms demonstrate that transparent PMMA substrates can be tracked over a substantial portion of the ToF sensor’s field of view, but that their presence introduces strong attenuation and interference effects as a function of insertion depth. Importantly, such behavior can only be resolved by analyzing histogram-level data; relying solely on the black-box distance outputs would conceal the dual-peak structure and the detailed evolution of signal degradation. These results emphasize the importance of raw-data access for material interaction studies and for developing correction strategies in practical FTIR–ToF integrations.

### 3.2. Setup with Dual-Sensor Layout

**Figure 5:**
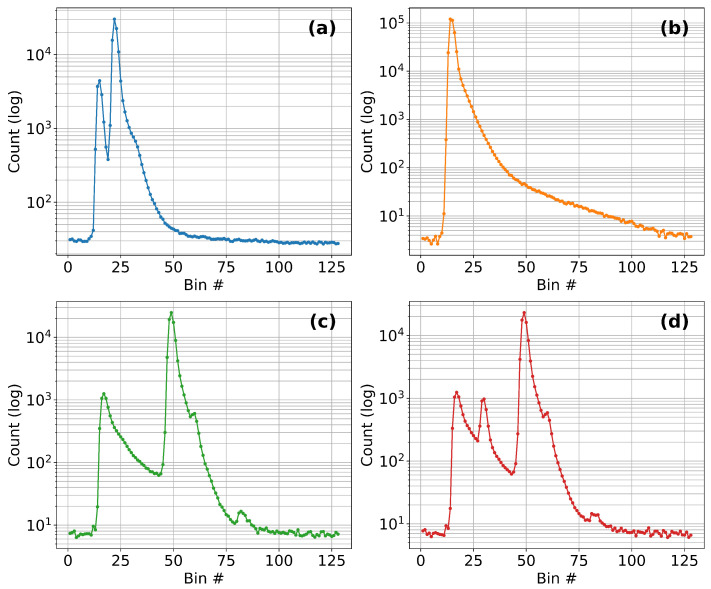
Histogram data of the dual-sensor setup with TMF8828 modules operating in FTIR (sensor Nr. 1) and proximity (sensor Nr. 2) configurations: (**a**) TMF8828 Nr. 2 baseline response without surface contact, showing distinct peaks corresponding to direct reflection and target return. (**b**) TMF8828 Nr. 2 response with a silicone contact directly above the module, where the near-field scattering dominates and saturates the signal, effectively “blinding” the sensor. (**c**) TMF8828 Nr. 1 baseline histogram response in FTIR configuration, showing characteristic dual-peak structure. (**d**) TMF8828 Nr. 1 response during surface contact with a silicone target, where scattering inside the light guide produces additional peaks at bin 32 and altered intensity distribution.

This subsection analyzes the behavior of the dual-sensor system under two conditions: detection of an elevated object positioned 120 mm above the PMMA light guide and a direct surface touch event. Figure 5a presents the histogram response of TMF8828 sensor Nr. 2 configured for proximity sensing. The first peak, consistently observed around bin 15, originates from inherent sensor crosstalk and internal reflections, while the second peak at bin 23 corresponds to the elevated object at 120 mm distance. The measured distance remains stable and unaffected while the PMMA light guide is positioned within the “blind spot” of the sensor, as described in the previous subsection. When the object makes direct contact with the surface above sensor Nr. 2 (Figure 5b), the second peak merges with the crosstalk peak, producing a saturated response at bin 15. This behavior indicates overexposure of the SPAD array, effectively “blinding” the proximity sensor to external objects under surface-touch conditions.

The baseline histogram of TMF8828 sensor Nr. 1, configured for FTIR-based tactile sensing, is shown in Figure 5c. Two dominant peaks are visible: the first at bin 15, attributed to sensor crosstalk and reflections from the adjacent PMMA edge, and the second at bin 48, arising from reflections at the opposite edge of the light guide. When the object establishes surface contact, a strong additional peak emerges at bin 32 (Figure 5d), corresponding to scattering of the guided light at the contact point. Importantly, no measurable interference or coupling is observed between the tactile and proximity sensors during the surface touch event.

These findings demonstrate that crosstalk between the two TMF8828 modules is negligible under normal operating conditions and occurs only in the presence of strong scattering events, such as direct contact. This confirms the feasibility of combining FTIR-based tactile sensing with ToF proximity detection in a dual-sensor configuration, enabling concurrent operation without significant performance degradation.

### 3.3. Analysis of Touch Reconstruction with Dual-Sensor Layout

The experimental results demonstrate that the TMF8828 ToF sensor, integrated with a PMMA light guide, reliably detects surface contact events through the mechanism of frustrated total internal reflection (FTIR). To evaluate multi-sensor operation, an additional TMF8828 module was placed beneath the PMMA light guide and configured for proximity detection, providing complementary free-space measurements to the FTIR-based tactile channel. It is important to note that all reconstructed images and heat maps shown in this subsection are computed exclusively from the histogram data of sensor Nr. 1 (FTIR configuration); the proximity sensor (Nr. 2) is used only as a source of optical excitation and for distance measurements, but its data are not used to form the tactile maps.

**Figure 6:**
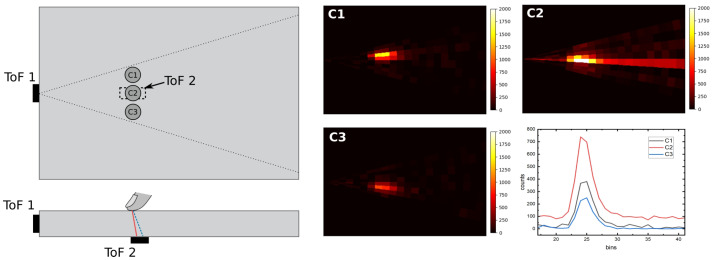
Experimental setup and sensor responses for the three measurement points C1, C2, and C3. (**Left**) Schematic representation of the dual-sensor arrangement, where ToF 1 is configured for FTIR-based tactile sensing and ToF 2 for proximity detection. (**Right**) Heat maps of the reconstructed tactile response for C1, C2, and C3, together with the corresponding histogram profiles. The color value in each pixel is obtained by processing the 8×8 multi-zone histograms of sensor Nr. 1: for each zone, the increase in photon counts in the touch-related bins is computed relative to a no-touch baseline and mapped to a 2D intensity image. Only data from the FTIR-configured sensor are used in this reconstruction; the proximity sensor contributes solely via optical coupling. The signal at C2 shows a localized increase in intensity and noise due to optical coupling between the two TMF8828 modules during surface contact. Despite this effect, ToF 1 reliably detects the surface touch via the FTIR mechanism across all positions.

Across all three measurement points, C1, C2, and C3 (as defined in Figure 6), a clear increase in signal intensity was observed by the FTIR-configured TMF8828 when the test object made contact with the light guide. This confirms that object contact consistently disrupts total internal reflection and enables accurate localization of the touch position.

A particularly notable finding was obtained at point C2, where the average signal intensity was approximately 85% higher than at C1 and C3. This amplification is attributed to localized optical coupling between the two TMF8828 modules: when the object touches directly above the proximity sensor at C2, scattered infrared light from that sensor partially couples into the light guide, boosting the amplitude of the FTIR signal. Although this effect slightly increases noise at C2, it is spatially localized, disappears once the object is removed, and does not compromise the overall fidelity of touch point reconstruction.

Despite the additional optical input observed at C2, the TMF8828 sensor remained fully operational and preserved sufficient signal integrity to correctly localize the contact point. This demonstrates the robustness of the FTIR-based sensing approach to moderate levels of optical interference, which is essential for practical applications where multiple sensors may operate in close proximity. Nevertheless, the elevated noise floor and amplified signal response at C2 underline potential limitations for scalability, particularly when integrating additional proximity sensors beneath the PMMA sheet. To address these challenges, future implementations could incorporate strategies such as physical or optical shielding, temporal multiplexing, or wavelength-selective filtering. Moreover, further investigations are warranted to evaluate the angular dependence of this interference and to study the role of object material and geometry in shaping the resulting signal distortion.

**Figure 7:**
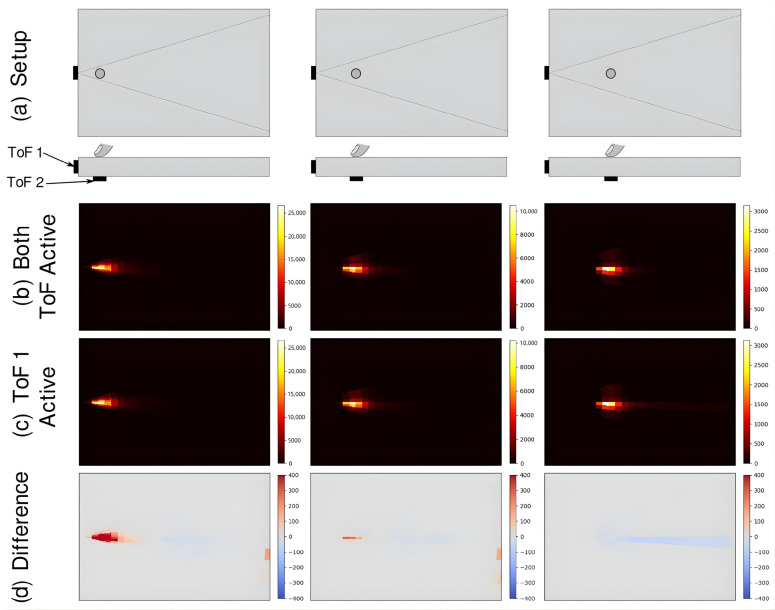
(**a**) Experimental setups with ToF 2 positioned at 3 cm, 6 cm, and 9 cm from ToF 1 (left to right). (**b**) Reconstructed tactile images with both ToF sensors active. (**c**) Reconstructed tactile images with only ToF 1 active. (**d**) Pixel-wise difference between panels (**b**,**c**).

Figure 7 investigates the influence of optical interference between two TMF8828 sensors when arranged in close proximity and operated under varying separation distances. Panel (d) shows the difference maps, which reveal that scattering artifacts introduced by ToF 2 diminish as its distance from ToF 1 increases. This indicates that the coupling between sensors is highly localized and decays rapidly with spatial separation.

At the same time, the reconstructed tactile images in panel (b) confirm that such scattering does not noticeably degrade the quality of FTIR-based contact detection. Even in configurations where both sensors are simultaneously active, the contact point remains sharply resolved. Furthermore, when no touch occurs directly above ToF 2, there is no measurable increase in background noise, demonstrating that the system can be scaled to larger sensor arrays without accumulating interference.

These results highlight the potential of combining multiple ToF modules in a distributed architecture to extend tactile coverage across larger substrates. The findings also emphasize that inter-sensor interference must be considered in system design but can be effectively mitigated through geometric arrangement and spatial separation.

From a performance perspective, the dual-sensor configuration achieves a spatial resolution of 10–12 mm for linear tactile input and an angular resolution of approximately 3.5°, enabling reliable localization of surface interactions. In parallel, the proximity channel provides distance measurements up to 2000 mm with an accuracy of ±1 mm, which meets the requirements of many collaborative robotics (COBOT) and human–machine interaction scenarios. Overall, the Optoskin architecture demonstrates a practical route to integrating tactile and proximity sensing in a compact, optically transparent platform.

In the present prototype we did not implement any synchronization between the two TMF8828 modules; both sensors operate continuously and simultaneously. Nevertheless, the available GPIO pins of the dToF devices could be used as synchronization lines to drive the emitters in separate time slots, effectively eliminating optical crosstalk at the expense of a reduced overall update rate for the dual-sensor system.

## 4. Conclusions

This study demonstrated the feasibility of integrating TMF8828 Time-of-Flight sensors with PMMA light guides for dual tactile–proximity sensing via frustrated total internal reflection (FTIR). Using histogram-level analysis, we systematically characterized how a transparent PMMA layer influences ToF measurements, identifying five distinct operating regions that reveal object “swaps,” signal decay, and recovery. These results highlight the unique capability of the TMF8828 to resolve both a transparent interface and a fixed background target—an advantage over simpler ToF devices that output only scalar distances.

We further examined dual-sensor configurations, showing that localized optical coupling between adjacent modules increases noise but does not compromise touch localization. This confirms the robustness of FTIR-based sensing under moderate interference and provides evidence that ToF arrays can be scaled to larger sensing surfaces. Importantly, the experiments demonstrate that both proximity and tactile functions can be achieved within a compact and transparent architecture.

It must be noted that the presented results are specific to 2 mm PMMA and a single ToF model. Variations in thickness, refractive index, scattering, and surface quality could significantly alter the observed behavior. Future work will, therefore, focus on extending the methodology to alternative substrates and optimized optical coupling strategies, and developing adaptive signal-processing algorithms for improved robustness in uncontrolled lighting environments.

This work establishes a novel proof-of-concept framework for FTIR–ToF integration, offering a promising foundation for next-generation tactile skins, transparent control panels, and human–robot interaction systems where simultaneous touch and proximity detection are required.

The rich histogram data and reconstruction pipeline presented in this work provide a suitable basis for training data-driven or AI/ML models, for example to classify contact versus proximity events or to compensate for crosstalk and material-dependent artifacts. Exploring such learning-based approaches will be an interesting direction for future work.

## Data Availability

The original contributions presented in this study are included in the article. Further inquiries can be directed to the corresponding author.
